# A systematic review and meta-analysis on the effect of virtual reality-based rehabilitation for people with Parkinson’s disease

**DOI:** 10.1186/s12984-023-01219-3

**Published:** 2023-07-20

**Authors:** Sun-Ho Kwon, Jae Kyung Park, Young Ho Koh

**Affiliations:** Division of Brain Disease Research, Department for Chronic Disease Convergence Research, Korea National Institute of Health, 187 Osongsaengmyeong2‑ro, Osong‑eup, Cheongju‑si, Chungcheongbuk‑do 28159 Republic of Korea

**Keywords:** Virtual reality, Parkinson’s disease, Balance, Gait, Systematic review, Meta-analysis

## Abstract

**Background:**

Virtual reality (VR) is a promising solution for individuals with Parkinson’s disease (PD) who experience symptoms that affect their daily activities and independence. Through VR-based rehabilitation, patients can improve their motor skills in a safe and stress-free environment, making it an attractive alternative to traditional in-person rehabilitation during the COVID-19 pandemic. This study aimed to provide the most recent and convincing evidence on the rehabilitative effects of VR technology compared with conventional treatments.

**Methods:**

Two investigators systematically searched Embase, MEDLINE, CINAHL, PEDro, and the Cochrane Library from their inception until May 31, 2022, to identify randomized controlled trials (RCTs) comparing the effectiveness of VR training with that of conventional treatment for patients with PD. Studies were selected based on the patient, intervention, comparator, and outcome criteria and assessed for the risk of bias using the Cochrane tool. Meta-analysis was conducted by pooling mean differences with 95% confidence intervals.

**Results:**

A total of 14 RCTs, involving 524 participants, were included in the meta-analysis. The results indicated that VR-based rehabilitation significantly improved balance function, as measured using the Berg balance scale (BBS) and activities-specific balance confidence. However, no statistically significant differences in gait ability, activities of daily living, motor function, and quality of life were observed between the experimental and control groups. Subgroup analysis revealed that combination therapy affected heterogeneity in the BBS analysis. Meta-regression analysis demonstrated a significant positive relationship, indicating that more recent studies have shown greater improvements in balance function.

**Conclusion:**

This study’s findings suggest that VR-based rehabilitation is a promising intervention for improving balance function in patients for PD compared with conventional treatment, and recent research supports its efficacy. However, future research should focus on conducting long-term follow-up studies and developing standardized protocols to comprehensively establish this intervention’s potential benefits.

**Supplementary Information:**

The online version contains supplementary material available at 10.1186/s12984-023-01219-3.

## Background

Patients with Parkinson’s disease (PD) often experience tremors, balance problems, and decreased motor abilities, which can significantly affect their daily activities [1]. These symptoms can negatively impact their quality of life and independence [2]. One potential solution to these challenges is virtual reality (VR) [3].

VR technology is a computer technology that simulates an environment similar to that of the real world. This technology allows users to interact in a three-dimensional (3D) virtual environment and experience various situations. Users can easily experience difficult situations in the real world using VR technology [4]. Depending on the degree of immersion, there are three types VR: non-immersive, semi-immersive, and fully immersive VR. Non-immersive reality is a type of VR where you can interact with the virtual environment through a computer screen, possibly a video game console, or an interface device. Semi-immersive VR primarily involves the use of large screens or projections to visually experience virtual 3D spaces. Fully-immersive VR involves the use of advanced wearable devices, such as head-mounted displays (HMDs), to enable users to actively participate in movement and interaction within virtual spaces, thereby experiencing a high level of presence and realism. All types of VR enable users to experience physically challenging situations in a safe environment, which can be particularly helpful in the rehabilitation of neurological conditions such as PD [5].

VR-based rehabilitation typically involves moving and performing actions in a virtual environment. These actions are implemented through computer simulations, and patients can improve their motor skills by identifying targets, following paths, and performing daily life movements in 3D virtual spaces [6]. Compared with previous rehabilitation methods, VR technology has several advantages. First, it allows users to safely and effectively experience dangerous or impossible situations in real environments. Second, in a virtual environment, patients can exercise at their own pace and difficulty level without experiencing stress. Third, VR technology can help patients build confidence in dealing with difficult real-world situations.

The COVID-19 pandemic has caused significant disruptions in healthcare delivery, including rehabilitation services [7]. Patients with PD are particularly vulnerable to the negative effect of social distancing and isolation, which can lead to decreased physical activity and worsening symptoms [8]. Home-based VR refers to the utilization of VR technology in the comfort and convenience of patients’ own home for rehabilitation. It allows patients to engage in therapy and exercises remotely, eliminating the need for frequent visits to hospitals or rehabilitation facilities. This approach enables patients to rehabilitate remotely, from the comfort and safety of their homes, guided by medical professionals and therapists [9, 10].

VR-based rehabilitation has shown promising results in improving motor function, balance, gait, and ADL in patients with neurological and musculoskeletal disorders. It is an effective treatment option for stroke, multiple sclerosis, and traumatic brain injury [11–13]. Several recent studies have shown that VR-based rehabilitation can also effectively improve balance and gait of patients with PD [14]. Furthermore, some interventions, including customized VR, have demonstrated efficacy in enhancing cognitive abilities such as attention and memory [15–17]. This can be a beneficial intervention for improving non-motor symptoms in patients with PD, such as PD-mild cognitive impairment or PD dementia.

Nonetheless, from a comprehensive perspective, the RCT results were inconsistent, with some showing significant improvement and others showing no difference compared with traditional therapy. We therefore conduct a systematic review of this field to present the latest trends and prospects. This systematic review and meta-analysis aimed to provide the most recent and convincing evidence on the rehabilitative effects of VR technology compared with conventional treatments.

## Methods

### Registration and search strategy

This study was conducted in compliance with the PRISMA guidelines [18]. The study protocol was registered under number CRD42022310868 in PROSPERO. A thorough search of the Embase, MEDLINE, CINAHL, PEDro, and Cochrane Library databases from their inception to May 31, 2022, was performed. The main search terms include Parkinson’s disease (“Parkinson’s disease” or “Parkinson*”) and virtual reality (“virtual reality,” “VR,” “game,” “gaming,” or “exergam*”). Boolean operators were used for combining the search terms. The literature search strategy in each database is presented in Additional file 1: Table S1.

### Study selection

The selection was performed independently by two reviewers (KSH and PJK) based on predetermined criteria. Disagreements were resolved through discussion or arbitration involving a third author (KYH) until a consensus was obtained. In accordance with the patient, intervention, comparator, and outcome (PICO) criteria, studies were included if they met the following inclusion criteria: (1) patients clinically diagnosed with PD without any limitations on sex, age, and disease duration or severity, (2) interventions including VR training compared with conventional treatment; and (3) randomized controlled trials (RCTs). VR training was defined as an intervention that uses specially programmed computers, visual immersion devices, and simulated experiences in artificially created environments to help improve or maintain patients’ physical and cognitive functions [5]. Conventional treatments, which are used as a control group in studies for comparison with VR training, include various physical therapies such as muscle strengthening and flexibility exercises, balance training, treadmill training, and functional training [19]. There were no limitations on the type or strategy of intervention in the experimental and control groups. The inclusion and exclusion criteria are detailed in Additional file 2: Table S2.

### Data extraction and quality assessment

The following data were extracted by two of the authors (KSH and PJK): (1) study characteristics: author, publication year and country; (2) sample characteristics for each study: sex, age, Hoehn and Yahr stage, intervention, protocol, supervision, related outcome and *P* value; (3) primary outcomes: balance function and gait ability; and (4) secondary outcomes: activities of daily living (ADL), motor function, and quality of life. Among the studies published by the same research team, those with overlapping research periods and participants were excluded.

Two authors (KSH and PJK) independently assessed the risk of bias in the included studies. We used version 2 of the Cochrane risk of bias tool for randomized trials (RoB2) to assess the risk of bias in the RCTs included in this systematic review [20]. The criteria included the following: (1) randomization process, (2) deviations from the intended intervention, (3) missing outcome data, (4) measurement of the outcome, and (5) selection of reported results. Each criterion was judged as ‘low risk of bias,’ ‘some concerns,’ or ‘high risk of bias.’

### Statistical analysis

Meta-analyses were conducted using the Review Manager 5.4 software (Cochrane Collaboration, Oxford, UK). For continuous variables, the inverse variance method was used to pool mean differences (MDs) with 95% confidence intervals (CIs). If the median and range were reported instead of mean and standard deviation (SD), these values were estimated using the methods developed by Luo et al*.* and Wan et al. [21, 22]. Heterogeneity among studies was assessed using the Q test and *I*^2^ statistic. *I*^2^ statistic was interpreted in accordance with the Cochrane Handbook for Systematic Reviews of Interventions. If the Cochrane Q statistical *P* value was < 0.10 and the *I*^2^ statistic was > 50%, significant heterogeneity was considered present, and a random-effects model was used. If otherwise, a fixed effects model was used [23]. A meta-regression analysis was performed using the publication year as the predictor variable to investigate whether the effect size of the studies included in the present meta-analysis varied as a function of the publication year. A mixed-effects model with publication year as the only covariate was fitted using the R package meta. To assess publication bias for asymmetry in the funnel plot, we performed Egger’s regression test. The intercept and slope of the regression line were estimated, and statistical significance was set at *P* < 0.1.

## Results

### Study characteristics

**Fig. 1:**
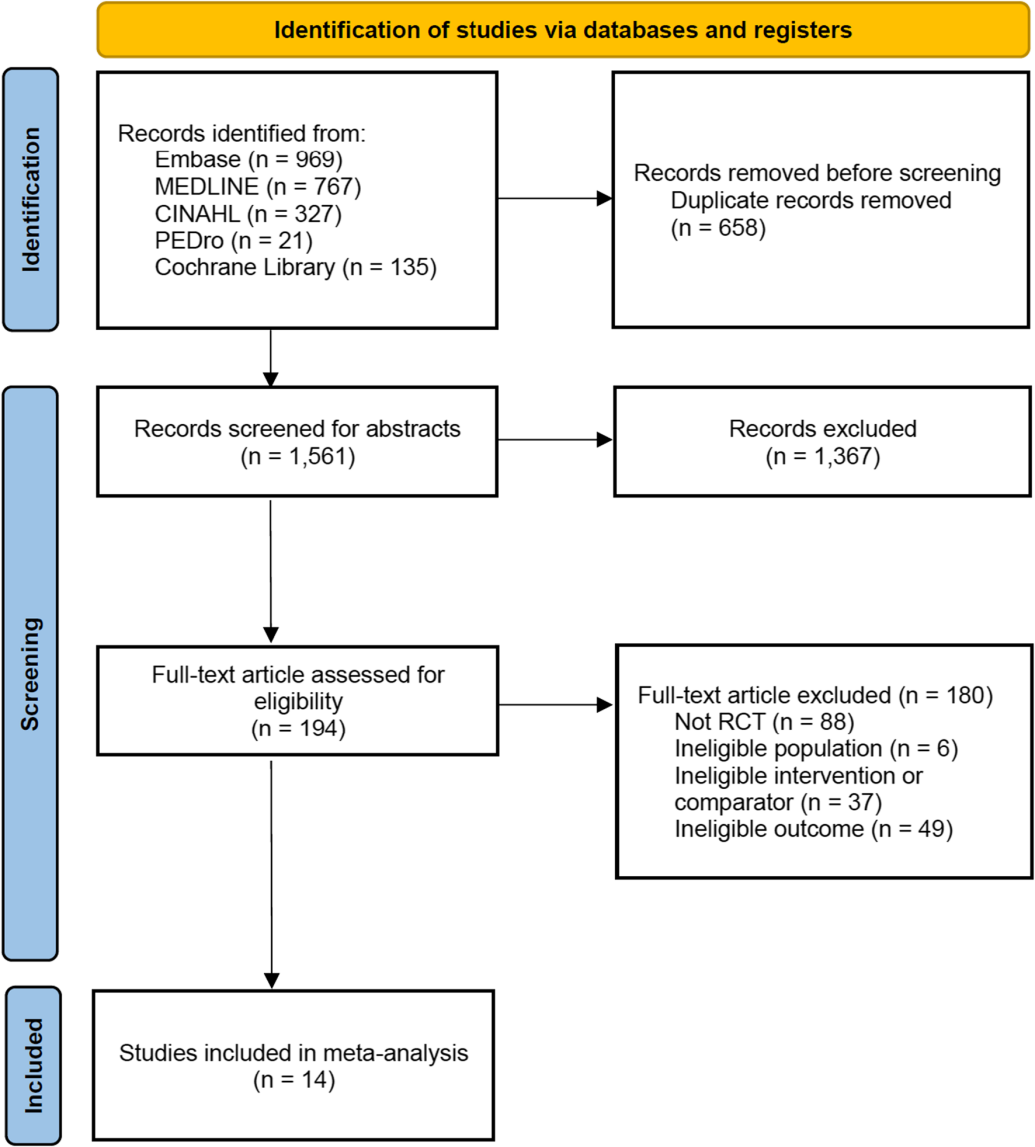
PRISMA diagram of the search strategy. A systematic search was performed across multiple databases including Embase, MEDLINE, CINAHL, PEDro, and Cochrane Library. After removing duplicate articles, excluding articles based on language and article type after reviewing abstracts, and conducting full-text screening, a final set of 14 randomized controlled trials (RCTs) met all the inclusion criteria

**Fig. 2:**
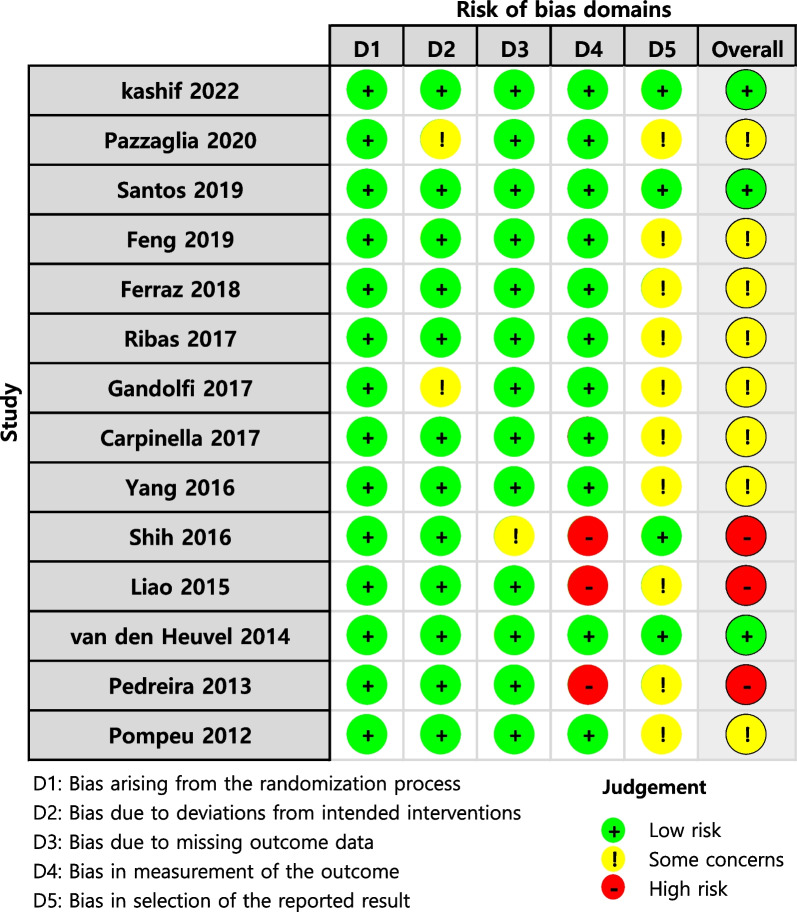
Assessment of risk of bias. Risk of bias assessment using Cochrane risk of bias 2 (RoB 2) tool. In this color-coded ranking, green color represents low risk of bias, yellow some concerns, and red high risk of bias

A total of 2219 studies were identified through the literature search (Fig. 1). After excluding 658 duplicate articles, 1367 articles were excluded based on language and article type after reviewing the abstracts. The remaining articles underwent full-text screening to ensure that they met the inclusion criteria, which resulted in the exclusion of additional 180 articles. Finally, 14 RCTs met all the inclusion criteria [24–37]. Participants’ demographics, interventions, and related outcome measures are presented in Table 1. The characteristics of intervention are detailed in Additional file 3: Table S3. The criteria used to assess the risk of bias in the included studies were based on RoB2 (Fig. 2). In summary, three studies were assessed as having a low risk of bias, eight studies were judged to raise some concerns, and three studies were deemed to have a high risk of bias.

**Table 1:** Main characteristics of included studies

Study (year)	Country	Sex (male/female)		Age		Hoehn and Yahr stage		Intervention		Intervention protocol	Supervision, supervisior	Related outcome measures	*P* value
		EG	CG	EG	CG	EG	CG	EG	CG				
Kashif (2022) [24]	Pakistan	13/9	12/10	63.86 ± 4.57	62.32 ± 4.61	2.11 ± 0.74	2.25 ± 0.67	Physical therapy + virtual reality (Nintendo Wii) + motor imagery	Physical therapy	60 min/d 3 d/wk 6, 12 wks Follow-up to 16 wks	Full, physical therapist	UPDRS II	< 0.001
												UPDRS III	< 0.001
												BBS	< 0.001
												ABC	< 0.001
Pazzaglia (2020) [25]	Italy	18/7	17/9	72 ± 7	70 ± 10	NR	NR	Virtual reality (NIRVANA)	Physical therapy	40 min/d 3 d/wk 6 wks	Full, physical therapist	BBS	NR
												DGI	NR
Santos (2019) [26]	Brazil	11/2	11/3	61.7 ± 7.3	64.5 ± 9.8	1.4 ± 0.6	1.3 ± 0.3	Virtual reality (Nintendo Wii)	Conventional exercise	50 min/d 2 d/wk 8 wks	Full, physiotherapist	BBS	0.968
												DGI	0.277
		9/5		66.6 ± 8.2		1.5 ± 0.4		Virtual reality (Nintendo Wii) + conventional exercise				TUGT	0.824
												PDQ-39	0.331
Feng (2019) [27]	China	8/7	9/6	67.47 ± 4.79	66.93 ± 4.64	3.03 ± 0.55	2.97 ± 0.58	Virtual reality	Physical therapy	45 min/d 5 d/wk 12 wks	Full, physical therapist	BBS	NR
												TUGT	NR
												UPDRS III	NR
												FGA	NR
Ferraz (2018) [28]	Brazil	10/10	16/6	67 (66–68)	71 (66–75)	2.5 (2.0–2.5)	2.5 (2.5–3.0)	Virtual reality (Xbox 360 with Kinect)	Functional training	50 min/d 3 d/wk 8 wks	Full, physiotherapist	6MWT	NS
												10MWT	NS
			11/9		67 (64–71)		2.5 (2.0–3.0)		Bicycle exercise				
												PDQ-39	NS
Ribas (2017) [29]	Brazil	4/6	4/6	61.70 ± 6.83	60.20 ± 11.29	1.25 (1–2)	1.5 (1–2)	Virtual reality (Nintendo Wii)	Conventional exercise	30 min/d 2 d/wk 12 wks 60d follow-up	Full, physiotherapist	BBS	0.043
												6MWT	0.023
Gandolfi (2017) [30]	Italy	23/15	28/10	67.45 (7.18)	69.84 (9.41)	2.5 (2.5–2.5)	2.5 (2.5–3.0)	Virtual reality (Nintendo Tele Wii)	Sensory integration balance training	50 min/d 3 d/wk 7 wks 1-month follow-up	Full, physiotherapist	BBS	0.02
												ABC	NS
												10MWT	NS
												DGI	NS
Carpinella (2017) [31]	Italy	14/3	9/11	73.0 ± 7.1	75.6 ± 8.2	2.7 ± 0.7	2.9 ± 0.5	Gamepad-based training	Physiotherapy	45 min/d 3 d/wk 7 wks 1-month follow-up	Full, physiotherapist	BBS	0.047
												10MWT	0.335
												TUGT	0.269
												UPDRS III	0.545
												ABC	0.186
												FOG-Q	0.695
												PDQ-39	0.844
Yang (2016) [32]	Taiwan	7/4	7/5	72.5 ± 8.4	75.4 ± 6.3	3(3–3)	3(3–3)	VR balance training	Conventional balance training	50 min/d 2 d/wk 6 wks Follow-up to 8 wks	Full, physiotherapist	BBS	0.893
												DGI	0.970
												PDQ-39	0.684
												UPDRS III	0.345
Shih (2016) [33]	Taiwan	9/1	7/3	67.5 ± 9.96	68.8 ± 9.67	1.6 ± 0.84	1.4 ± 0.52	Balance-based exergaming with Kinect	Conventional balance training	50 min/d 2 d/wk 8 wks	Not clear	BBS	NS
												TUGT	NS
Liao (2015) [34]	Taiwan	6/6	6/6	67.3 ± 7.1	65.1 ± 6.7	2.0 ± 0.7	2.0 ± 0.8	Virtual reality (Nintendo Wii)	Traditional exercise	45 min/d 2 d/wk 6 wks 1-month follow-up	Not clear	TUGT	< 0.01
												PDQ-39	< 0.01
van den Heuvel (2014) [35]	Netherland	12/5	8/8	66.3 ± 6.39	68.8 ± 9.68	2.5 (2.0–2.5)	2.5 (2.0–3.0)	Visual feedback training	Conventional balance training	60 min/d 2 d/wk 5 wks follow-up to 12 wks	Full, physical therapist	BBS	0.108
												10MWT	0.171
												UPDRS III	0.021
												PDQ-39	0.444
Pedreira (2013) [36]	Brazil	11/5	11/4	61.1 ± 8.2	66.2 ± 8.5	2.5 ± 0.6	2.4 ± 0.7	Virtual reality (Nintendo Wii)	Physical therapy	40 min/d 3 d/wk 4 wks	Not clear	PDQ-39	NR
Pompeu (2012) [37]	Brazil	NR	NR	67.4 ± 8.1	66.2 ± 8.3	1–2	1–2	Wii-based motor and cognitive training	Global and balance exercise	60 min/d 2 d/wk 7 wks 60d follow-up	Full, physiotherapist	UPDRS II	NS
												BBS	NS

*ABC* activities-specific balance confidence, *ADL* activities of daily living, *BBS* Berg balance scale, *CG* control group, *d* day, *DGI* dynamic gait index, *EG* experimental group, *FGA* functional gait assessment, *FOGQ* freezing of gait questionnaire, m minute, *Mini-BEST* mini balance evaluation system test, *NR* not reported, *NS* not significant, *PDQ-39* Parkinson’s disease questionnaire-39, *TUGT* time up and go test, *UPDRS* Unified Parkinson’s disease rating scale, *wk* week, *wks*, weeks, *6MWT* 6-min walk test, *10MWT* 10-m walk test

### Balance function

**Fig. 3:**
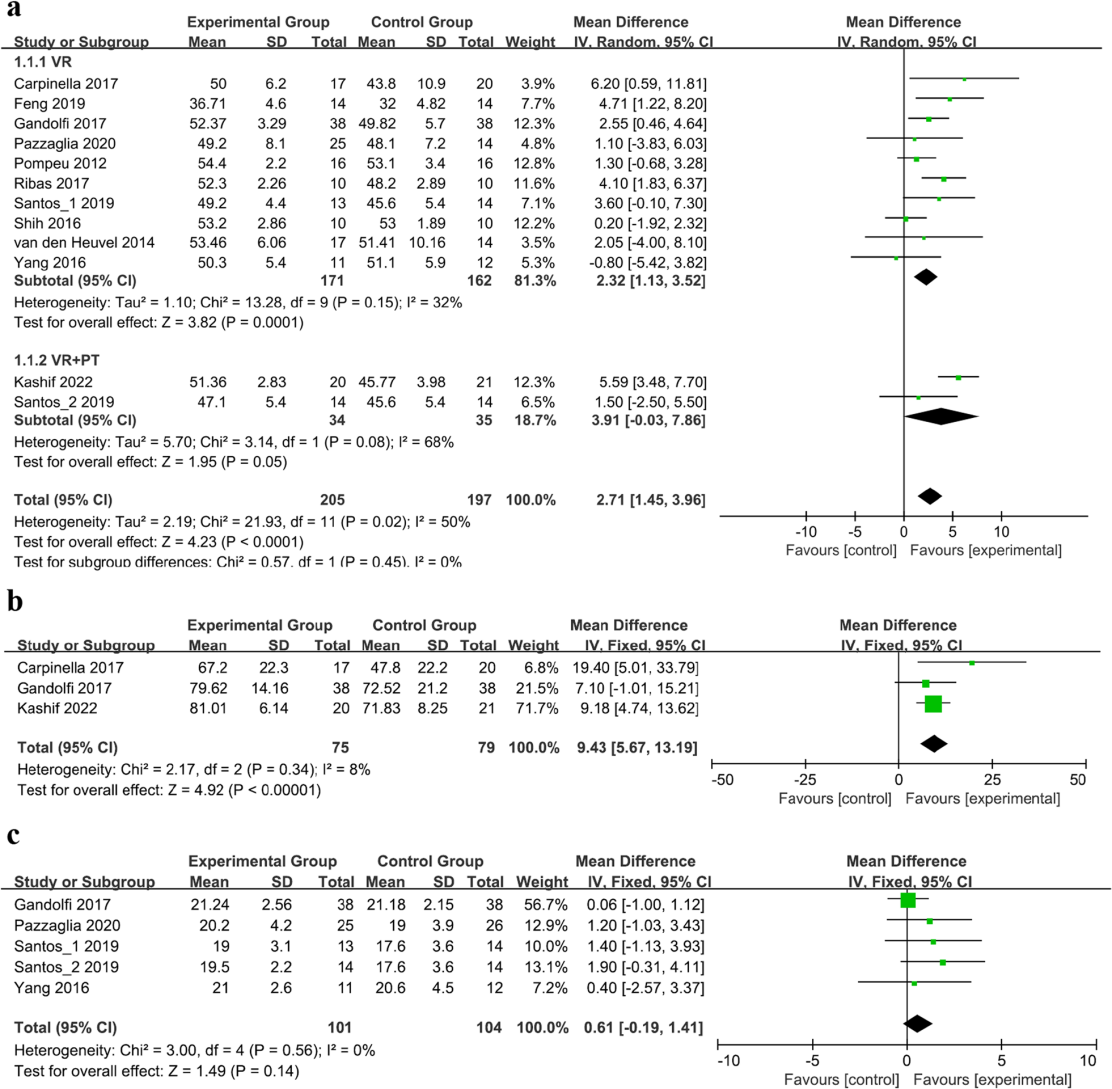
Forest plot for meta-analysis of primary outcomes related to balance function. Mean difference (95% CI) of the effect of VR-based rehabilitation (experimental group) compared with conventional treatment (control group) on **a** BBS, **b** ABC, and **c** DGI. *CI* confidence interval, *SD* standard deviation

12 studies comprising 388 patients assessed balance ability using the Berg balance scale (BBS) scores (Fig. 3a) [24–27, 29–33, 35, 37]. An analysis of these studies revealed significantly higher BBS scores in the experimental group compared with the control group (MD = 2.71, 95% CI = 1.45 to 3.96, *P* < 0.001), and moderate heterogeneity was observed (*I*^2^ = 50%, *P* = 0.02). To further explore this heterogeneity, subgroup analysis was performed based on whether or not combination therapy was administered, dividing the total group into VR and VR+PT subgroups. Heterogeneity was no longer significant in the VR subgroup (*I*^2^ = 32%, *P* = 0.15), whereas heterogeneity remained significant in the VR+PT subgroup (*I*^2^ = 68%, *P* = 0.08).

Three studies, involving 154 patients, evaluated balance confidence by measuring scores on the activities-specific balance confidence (ABC) scale (Fig. 3b) [24, 30, 31]. A significant increase in the ABC scale score was observed in the VR-based rehabilitation group compared with the control group (MD = 9.43, 95% CI = 5.67 to 13.19, *P* < 0.001). No heterogeneity was detected among the included studies (*I*^2^ = 8%, *P* = 0.34).

The balance performance during gait was measured using the dynamic gait index (DGI) in five studies involving 191 patients (Fig. 3c) [25, 26, 30, 32]. The pooled analysis revealed no statistically significant difference between the two groups (MD = 0.61, 95% CI = − 0.19 to 1.41, *P* = 0.14), and no heterogeneity was detected among the included studies (*I*^2^ = 0%, *P* = 0.56).

Finally, one study involving 28 patients investigated the effects of VR-based rehabilitation on postural stability and balance using the functional gait assessment (FGA) [27]. The results showed a significant improvement in the experimental group (*P* < 0.05).

### Gait ability

**Fig. 4:**
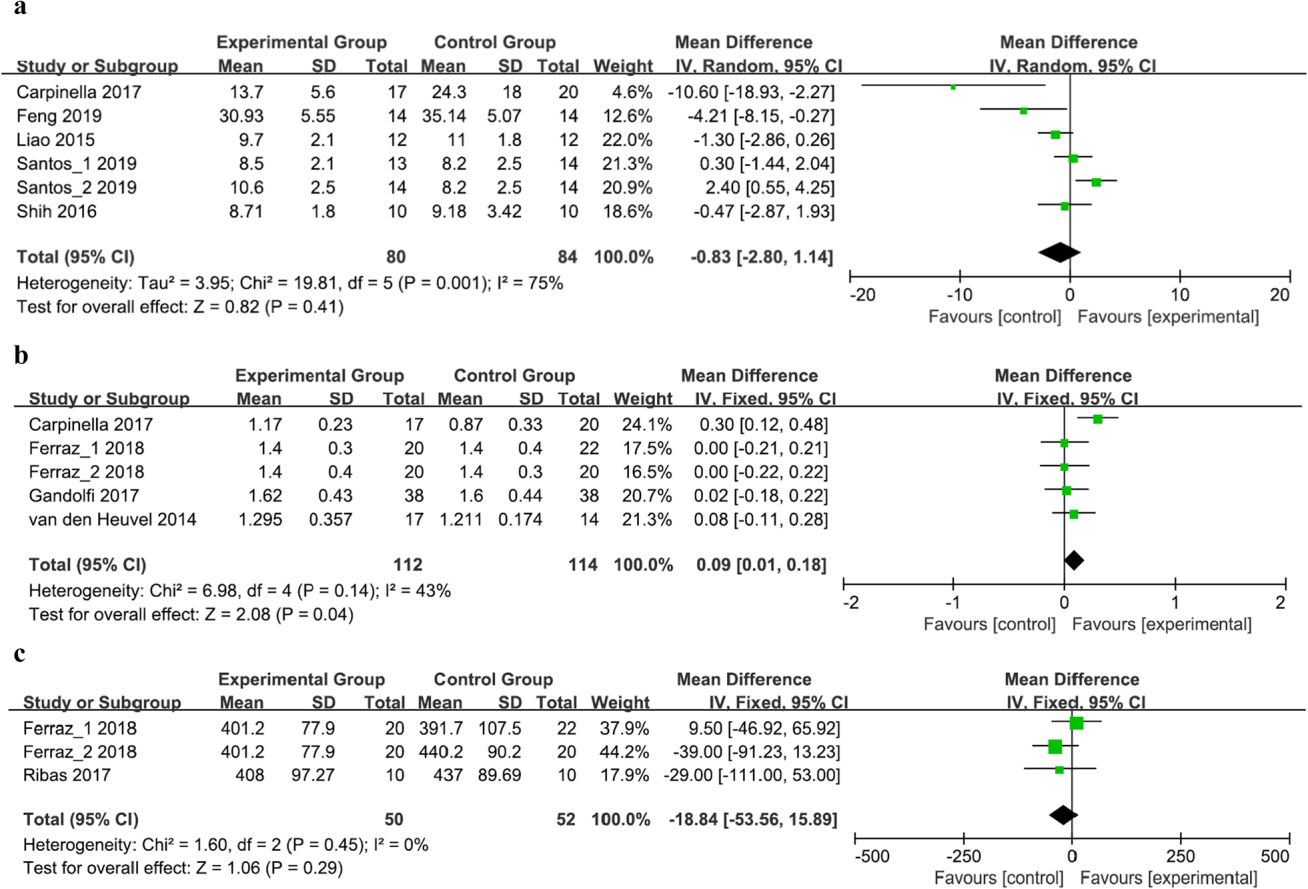
Forest plot for meta-analysis of primary outcomes related to gait ability. Mean difference (95% CI) of the effect of VR-based rehabilitation (experimental group) compared with conventional treatment (control group) on **a** TUGT, **b** 10MWT, and **c** 6MWT. *CI* confidence interval, *SD* standard deviation

Six studies involving 150 patients examined functional mobility using the time up and go test (TUGT) (Fig. 4a) [26, 27, 31, 33, 34]. The difference between the experimental and control groups was not statistically significant (MD = − 0.83, 95% CI = − 2.80 to 1.14, *P* = 0.41); however, heterogeneity was observed (*I*^2^ = 75%, *P* = 0.001).

The 10-m walk test (10MWT) was evaluated in five studies involving 206 patients (Fig. 4b) [28, 30, 31, 35], and the 6-min walk test (6MWT) was reported in three studies involving 82 patients (Fig. 4c) [28, 29]. The results showed a significant improvement in gait speed, as measured using the 10MWT, in the experimental group (MD = 0.09, 95% CI = 0.01 to 0.18, *P* = 0.04), whereas no change in walking capacity was observed using the 6MWT (MD = − 18.84, 95% CI = − 53.56 to 15.89, *P* = 0.29). The heterogeneity was insignificant (respectively, *I*^2^ = 43%, *P* = 0.14; *I*^2^ = 0%, *P* = 0.45).

Freezing severity was evaluated in one study involving 37 patients using the freezing of gait questionnaire (FOGQ) [31], and there was no statistically significant difference between the experimental and control groups (*P* = 0.695, Cohen’s d = − 0.13).

### Activities of daily living/motor function

**Fig. 5:**
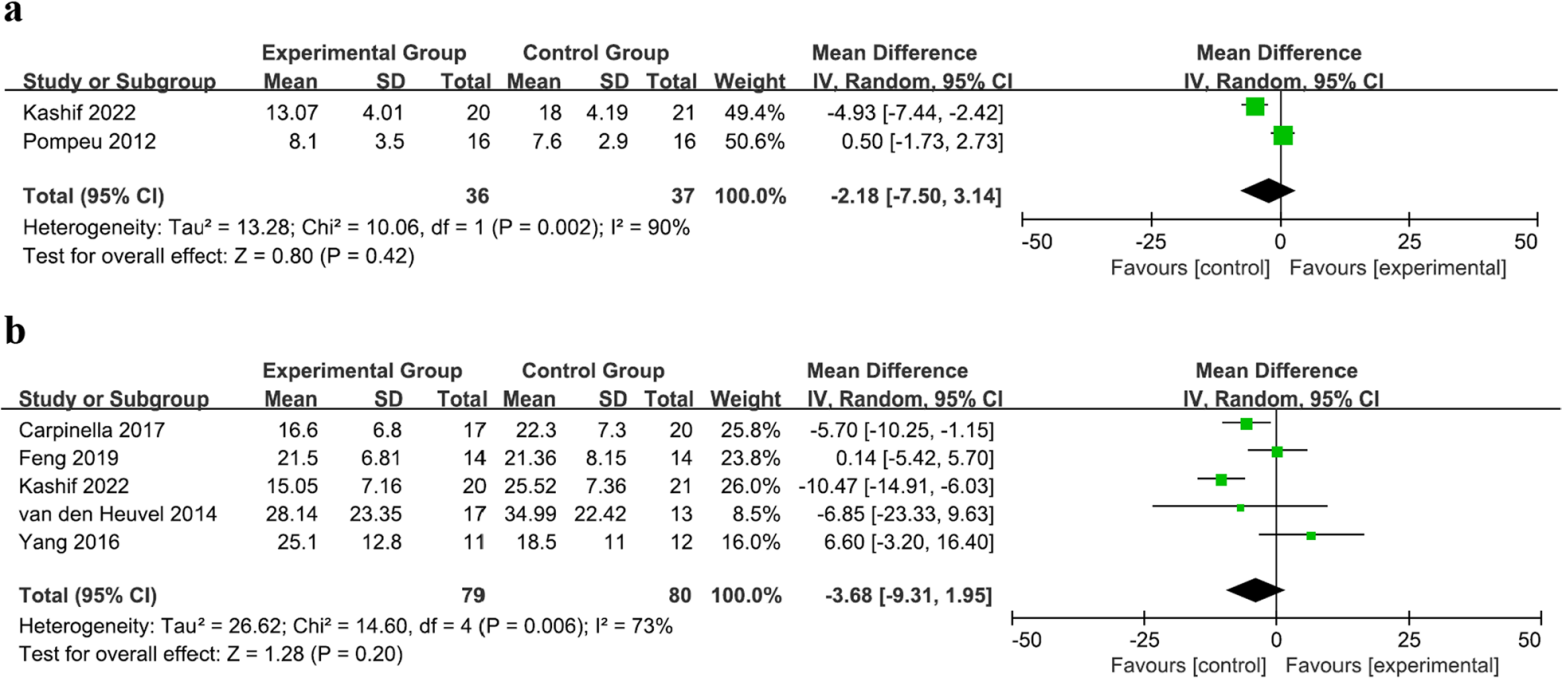
Forest plot for meta-analysis of secondary outcomes related to activities of daily living and motor function. Mean difference (95% CI) of the effect of VR-based rehabilitation (experimental group) compared with conventional treatment (control group) on **a** UPDRS II and **b** UPDRS III. *CI* confidence interval, *SD* standard deviation

Two studies involving 73 patients assessed the ADL using the Unified Parkinson’s disease rating scale (UPDRS) II (Fig. 5a) [24, 37]. The results showed no statistically significant difference between the two groups (MD = − 2.18, 95% CI = − 7.50 to 3.14, *P* = 0.42). High statistical heterogeneity was observed among the studies (*I*^2^ = 90%, *P* = 0.002).

Five studies involving 159 patients evaluated motor function using UPDRS III (Fig. 5b) [24, 27, 31, 32, 35]. The results revealed that the UPDRS III scores were equivalent between the two groups (MD = − 3.68, 95% CI = − 9.31 to 1.95, *P* = 0.20), and a substantial heterogeneity was observed (*I*^2^ = 73%, *P* = 0.006).

### Quality of life

**Fig. 6:**
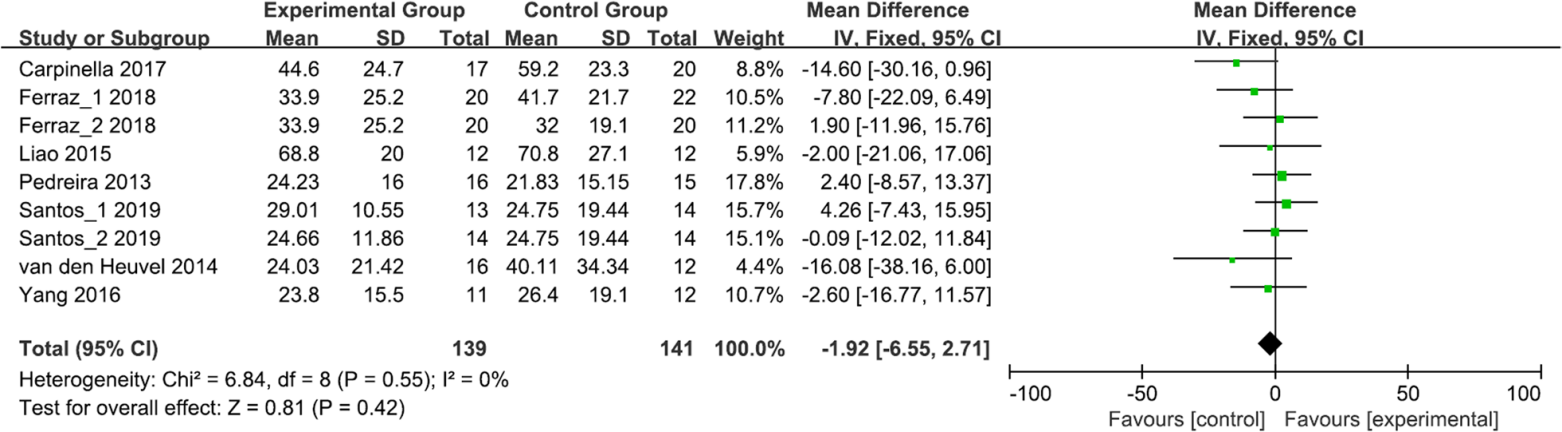
Forest plot for meta-analysis of secondary outcome related to quality of life. Mean difference (95% CI) of the effect of VR-based rehabilitation (experimental group) compared with conventional treatment (control group) on PDQ-39. *CI* confidence interval, *SD* standard deviation

Nine studies involving 146 patients investigated the effect of VR-based rehabilitation on the quality of life using the Parkinson’s disease questionnaire-39 (PDQ-39) (Fig. 6) [26, 28, 31, 32, 34–36]. The meta-analysis demonstrated no significant difference between the two groups (MD = − 1.92, 95% CI = − 6.55 to 2.71, *P* = 0.42), and no heterogeneity was observed among the included studies (*I*^2^ = 0%, *P* = 0.55).

### Meta-regression

**Fig. 7:**
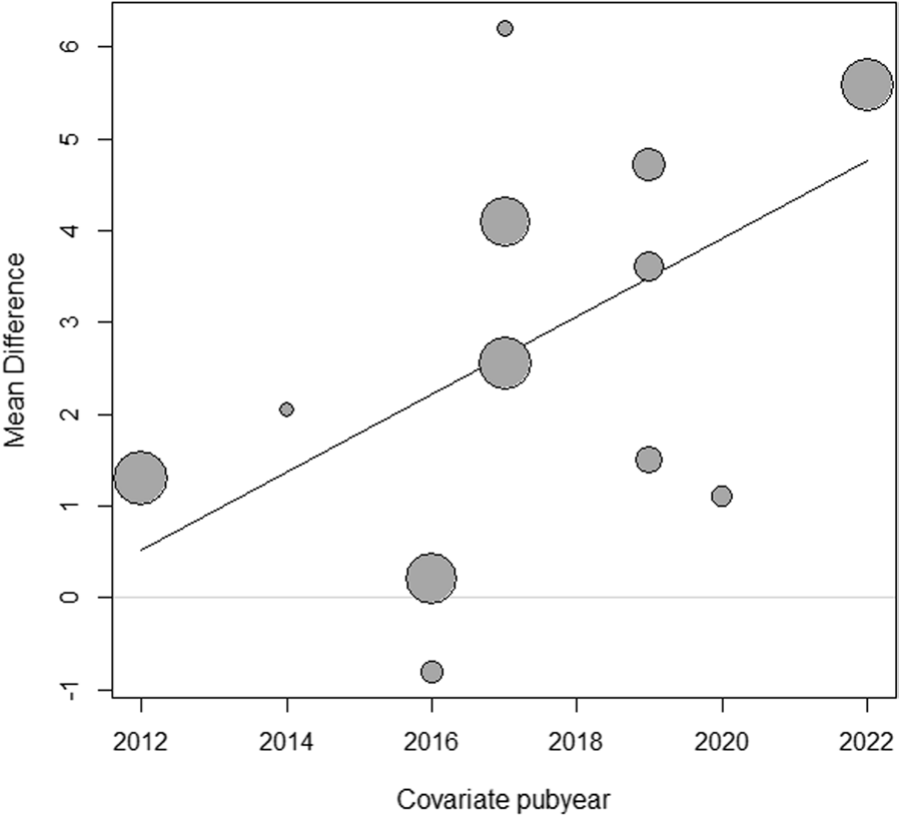
Meta-regression analysis based on publication year. Bubble plot of the relationship between publication year and mean difference in BBS scores

A meta-regression analysis was performed to examine the association between the publication year and the MD in BBS scores (Fig. 7) [24–27, 29–33, 35, 37]. The results indicated a significant positive relationship (β = 0.42, 95% CI = 0.06 to 0.79,* P* < 0.05), suggesting that the improvement in balance function varies depending on the publication year, with more recent studies showing greater improvement. Heterogeneity among the studies was low (*I*^2^ = 21.4%) after including the predictor, indicating no significant unexplained variability. The Q test for heterogeneity was insignificant (Q = 12.13, df = 10, *P* = 0.28), suggesting that the observed heterogeneity was likely due to chance.

### Publication bias

Visual assessment of the funnel plot showed that the studies were distributed symmetrically regarding BBS [24–27, 29–33, 35, 37], suggesting no publication bias in the present meta-analysis. Egger’s regression test (*P* = 0.99) confirmed the absence of potential publication bias. In addition, funnel plot analysis was performed for other functional outcomes such as balance function, gait ability, activities of daily living, motor function, and quality of life. However, it should be noted that the number of papers included in our study for these outcomes was less than 10, and as a result, publication bias cannot be reliably analyzed using the funnel plot analysis or Egger’s test. The results are presented in Additional file 4: Figs. S1, S2, S3, and S4.

## Discussion

This systematic review included 14 RCTs with 524 patients, comparing the effectiveness of VR-based rehabilitation to conventional interventions in patients with PD. The results provided valuable insights into the benefits of VR-based rehabilitation in improving balance function in patients with PD. Notably, meta-regression analysis revealed a positive relationship between the publication year and improvement in balance function, suggesting that more recent studies showed greater advancements in this field. On the other hand, in the meta-analysis, there was no statistically significant differences in gait ability, ADL, motor function, and quality of life. This indicates that current VR-based interventions are partially effective in improving symptoms, highlights the need for further technological advances focusing on gait and motor function to enhance patients’ overall quality of life.

One of the reasons why VR-based rehabilitation is particularly effective in improving balance function in patients with PD is because it challenges the patient’s balance through game-based VR program that incorporate activities requiring weight shifting and balance maintenance [24, 26, 29, 30, 37]. Incorporating game-like elements and other interactive features can increase patient enjoyment and willingness to participate in training sessions. This increased motivation can lead to better training outcomes and an overall improvement in balance. Another advantage of VR-based rehabilitation is that it can be tailored to individual patient needs. Customized training can help patients progress faster and more effectively than conventional treatments [31, 33, 35]. In addition, patients can observe their performance in real-time and receive immediate feedback regarding movement and balance control. This allows them to coordinate their movements and immediately improve their balance, leading to faster improvements in overall balance [38]. Therefore, VR-based rehabilitation effectively improves balance in patients with PD because it provides a high level of immersion, customized training, immediate feedback, and motivation through a game-like experience. These advantages make it a powerful tool for rehabilitation and a promising avenue for future research on PD treatment.

The significant association between the publication year and improvement in balance indicates that recent studies have developed more effective rehabilitation methods and measurement techniques than previous studies, which can increase the practical value of rehabilitation therapy. With advances in VR technology, it has become possible to create more realistic virtual environments, making VR a more suitable tool for rehabilitation. This technological development has enabled patients to experience virtual environments more realistically, thereby providing more effective rehabilitation therapy [39]. In addition, the increase in the accessibility of VR technology may be associated with the recent trend of its increased effectiveness [40, 41]. Previously, VR-based rehabilitation was limited because expensive equipment and specialized skills were needed. However, with recent technological advancements, VR-based rehabilitation equipment has become cheaper and easier to use, making it available to more patients [42]. This can increase patient engagement and participation, leading to more effective rehabilitation therapies. Therefore, VR-based rehabilitation treatments have recently shown more effective results.

VR-based rehabilitation has shown promising results in terms of clinical outcomes in patients with PD. However, much remains to be explored regarding the potential benefits of this therapy in different patient subgroups. For example, it may be valuable to investigate the effectiveness of VR-based rehabilitation in patients with varying disease severities, ages, and cognitive functions. Furthermore, future studies should focus on comparing the efficacy of VR-based rehabilitation with that of other treatment types, such as combination therapy with existing treatments or emerging technologies. Despite including some studies that investigated the combined use of VR-based rehabilitation and conventional treatment for PD, the interpretation of the results remains challenging owing to the limited number of studies available and the heterogeneity of the intervention protocols. Therefore, further studies with larger sample sizes and standardized protocols are warranted to fully elucidate the potential benefits of combining VR-based technologies with conventional treatments.

Regarding the safety of VR-based rehabilitation for patients with PD, the literature indicates that adverse events associated with VR are generally rare and mild. However, as the level of immersion in VR increases, there are additional considerations for ensuring safe rehabilitation, such as regular rest periods, securing a safe surrounding environment, and emotional stability. Particularly in rehabilitation, it is essential for instructors to educate users on proper usage techniques and safety procedures. The majority of studies analyzed in this systematic review utilized non-immersive VR technology (including one study using semi-immersive VR [25]) under the supervision of physical therapist during training sessions, and no significant adverse effects were reported or mentioned in each study. Additionally, two of the studies demonstrated the safety and effectiveness of remote rehabilitation by conducting home-based VR under supervision [30, 32]. It has been reported that even fully immersive VR can be considered safe for walking on the elderly and patients with PD [43]. Nevertheless, caution should be exercised regarding potential adverse effects such as cybersickness or falls that may occur when using HMDs [44–46].

RCTs are considered the gold standard for evaluating the efficacy of interventions. However, several limitations of RCTs on VR-based rehabilitation for patients with PD need to be addressed. First, blinding participants and therapists to treatment allocation is challenging in VR studies because it is difficult to blind participants to the type of therapy they receive. This can lead to a bias in the results because the participants may have had different expectations or preferences for each treatment arm. Second, conventional clinical outcome measures in VR studies may not accurately capture the specific changes in motor and non-motor symptoms that can be achieved with VR-based rehabilitation. Therefore, there is a need to develop new and specific outcome measures for VR-based rehabilitation for patients with PD. Finally, long-term follow-up assessments are needed in VR-based rehabilitation studies. Notably, some studies have reported positive outcomes immediately after VR-based rehabilitation; however, it is unclear whether these outcomes are maintained in the long term. Therefore, long-term follow-up assessments are required to determine the sustainability of VR-based rehabilitation.

## Conclusion

The results of this meta-analysis suggest that VR-based rehabilitation can effectively improve balance in patients with PD. Specifically, the experimental group demonstrated a significantly higher BBS and ABC scale scores than the control group. In addition, walking speed measured using 10MWT was significantly improved in the experimental group. However, no significant differences were observed in DGI, 6MWT, or UPDRS II and III scores. Meta-regression analysis suggested that the improvement in balance function varies depending on the publication year, with more recent studies showing greater improvement. Overall, these results indicate that VR-based rehabilitation programs may have a positive effect on balance function in patients with PD, and further studies are needed to determine the optimal VR intervention for these patients.

## Supplementary Information

**Additional file 1: Table S1.** Literature search strategy in each databases.

**Additional file 2: Table S2.** Details on inclusion and exclusion criteria.

**Additional file 3: Table S3.** Intervention characteristics of included studies.

**Additional file 4: Figure S1.** Funnel plots for meta-analysis of balance function. **Figure S2.** Funnel plots for meta-analysis of gait ability. **Figure S3.** Funnel plots for meta-analysis of activities of daily living and motor function. **Figure S4.** Funnel plot for meta-analysis of quality of life.

## Data Availability

The datasets supporting the conclusions of this article are included within the article.

## Abbreviations

PD: Parkinson’s disease
VR: Virtual reality
3D: Three-dimensional
RCTs: Randomized controlled trials
BBS: Berg balance scale
ABC: Activities-specific balance confidence
DGI: Dynamic gait index
FGA: Functional gait assessment
6MWT: 6-Minute walk test
10MWT: 10-Meter walk test
TUGT: Time up and go test
FOGQ: Freezing of gait questionnaire
ADL: Activities of daily living
UPDRS: Unified Parkinson’s disease rating scale
PDQ-39: Parkinson’s disease questionnaire-39
RoB2: Version 2 of the Cochrane risk of bias tool for randomized trials
MD: Mean difference
CI: Confidence interval
