# Supplementary material for: A systematic review and meta-analysis on the effect of virtual reality-based rehabilitation for people with Parkinson’s disease
Source: J Neuroeng Rehabil. 2023 Jul 20;20:94. doi: 10.1186/s12984-023-01219-3 (PMC10360300; doi:10.1186/s12984-023-01219-3)
Supplement: Supplementary file 1 — Additional file 1: Table S1. Literature search strategy in each databases. [file 12984_2023_1219_MOESM1_ESM.docx]

**Table S1.** Literature search strategy in each database

| **Database** |  | **Search** | **Search Terms** |
| --- | --- | --- | --- |
| Embase | Patient | #1 | exp Parkinson’s disease/ |
|  |  | #2 | Parkinson*.mp. |
|  |  | #3 | #1 OR #2 |
|  | Intervention | #4 | exp virtual reality/ |
|  |  | #5 | VR.mp. |
|  |  | #6 | exp game/ |
|  |  | #7 | exp gaming/ |
|  |  | #8 | exergam*.mp. |
|  |  | #9 | OR/#4-#8 |
|  | Total | #10 | #3 AND #9 |
| MEDLINE | Patient | #1 | exp Parkinson’s disease/ |
|  |  | #2 | Parkinson*.mp. |
|  |  | #3 | #1 OR #2 |
|  | Intervention | #4 | exp virtual reality/ |
|  |  | #5 | VR.mp. |
|  |  | #6 | exp game/ |
|  |  | #7 | exp gaming/ |
|  |  | #8 | exergam*.mp. |
|  |  | #9 | OR/#4-#8 |
|  | Total | #10 | #3 AND #9 |
| CINAHL | Patient | S1 | Parkinson's disease OR Parkinson* |
|  | Intervention | S2 | virtual reality OR vr OR game OR gaming OR exergam* |
|  | Total | S3 | S1 AND S2 |
| PEDro | Total | Simple search | parkinson's disease, virtual reality |
| Cochrane | Patient | 1 | “Parkinson disease” |
|  | Intervention | 2 | “virtual reality” |
|  | Total | 3 | 1 AND 2 |
