# Supplementary material for: A systematic review and meta-analysis on the effect of virtual reality-based rehabilitation for people with Parkinson’s disease
Source: J Neuroeng Rehabil. 2023 Jul 20;20:94. doi: 10.1186/s12984-023-01219-3 (PMC10360300; doi:10.1186/s12984-023-01219-3)
Supplement: Supplementary file 2 — Additional file 2: Table S2. Details on inclusion and exclusion criteria. [file 12984_2023_1219_MOESM2_ESM.docx]

**Table S2.** Systematic review inclusion and exclusion criteria

| **Category** | **Inclusion criteria** | **Exclusion criteria** |
| --- | --- | --- |
| Patients | - Patients clinically diagnosed with Parkinson’s disease without any limitations on sex, age, and disease duration or severity | - Diseases/conditions other than Parkinson’s disease |
| Interventions | - Interventions including virtual reality (VR) training compared with conventional treatment | - Not VR-based interventions - Non-conventional treatments for Parkinson’s disease |
| Outcomes | - Primary outcomes: balance function, gait ability - Secondary outcomes: activities daily living, motor function, quality of life | - Outcomes not relevant - Analysis based on the same set of data |
| Publication language | - English | - Any other language |
| Study  design | - Randomized controlled trials (RCTs) | - Non-RCTs (case reports, case-control studies, observational studies) - Preclinical trials, animal studies |
| Publication format | - Peer-reviewed research articles | - Books, Reviews, letters, editorials, opinions, conference abstracts, proceedings |
