# Supplementary material for: A systematic review and meta-analysis on the effect of virtual reality-based rehabilitation for people with Parkinson’s disease
Source: J Neuroeng Rehabil. 2023 Jul 20;20:94. doi: 10.1186/s12984-023-01219-3 (PMC10360300; doi:10.1186/s12984-023-01219-3)
Supplement: Supplementary file 3 — Additional file 3: Table S3. Intervention characteristics of included studies. [file 12984_2023_1219_MOESM3_ESM.docx]

**Table S3.** Intervention characteristics of included studies.

| **Study**  **(Year)** | **Intervention** | | **Types of Training** | **Protocol** |
| --- | --- | --- | --- | --- |
| Kashif  (2022) | Experimental group | Physical therapy + Virtual reality (Nintendo Wii) + Motor imagery | - PT (40 min) : warm-up exercises, Stretching exercises, Strengthening exercises, relaxation exercises - VR (10-15 min) : tennis, bowling, boxing, kicking, table tilt, penguin slide, title city, soccer, torso twist, single leg stance - MI (5-10 min) : watching the recorded videos, relaxing, performing activities | 60 minutes/day  3 days/week  6, 12 weeks,  follow-up to 16 weeks |
|  | Control group | Physical therapy | - PT (60 min) : warm-up exercises, Stretching exercises, Strengthening exercises, relaxation exercises, walking and cycling |  |
| Pazzaglia  (2020) | Experimental group | Virtual reality | - VR (40 min) : exercise 1 (to touch a moving trumpet), exercise 2 (to touch a rose), exercise 3 (to lead a dog), exercise 4 (to touch eggs), exercise 5 (to reach a mole), exercise 6 (to perform a motor task), exercise 7 (to clear all the leaves) | 40 minutes/day  3 days/week  6 weeks |
|  | Control group | Physical therapy | PT (40 min) : warm-up phase (passive mobilization of main joints and muscular strengthening of lower limbs), active phase (exercises of motor coordination with upper and lower limbs, balance training, start and stop exercises, and walking training), cool-down phase (manipulation exercises, mobilization exercises and respiratory exercises) |  |
| Santos  (2019) | Experimental group_1 | Virtual reality (Nintendo Wii) | - Stretching (10 min) - VR (40 min) : boxing, soccer heading, golf, running | 50 minutes/day  2 days/week  8 weeks |
|  | Experimental group_2 | Virtual reality (Nintendo Wii) + Conventional exercise | - Stretching (10 min) - VR (20 min) - PT (20 min) |  |
|  | Control group | Conventional exercise | Stretching (10 min)PT (40 min) : diagonal, active assisted and resisted active movements, based on the proprioceptive neuromuscular facilitation (PNF) patterns in the upper limbs, scapula, pelvis and lower limbs |  |
| Feng  (2019) | Experimental group | Virtual reality | - VR (45 min) : warm-up, hands and feet touch the ball, hard boating, take the maze, cool-down | 45 minutes/day  5 days/week  12 weeks |
|  | Control group | Physical therapy | - PT (45 min) : warm-up, balance, physical condition, coordination, cool-down |  |
| Ferraz  (2018) | Experimental group | Virtual reality  (Xbox 360 with Kinect) | - VR (50 min) : river rush, reflex ridge, 20,000 leaks | 50 minutes/day  3 days/week  8 weeks |
|  | Control group_1 | Functional training | - FT (50 min) : gait with obstacles, going up and down stairs and ramp, sitting and standing exercises, side gears, balance exercise in proprioceptive platform, activities with balls, step exercises, foot tip exercises, graded reaching activities, gait training |  |
|  | Control group_2 | Bicycle exercise | - BE (50 min) : aerobic training on a stationary bicycle (in the 1^st^ wk 50% of maximum heart rate, in the 2^nd^ and 3^rd^ wk 55%, in the 4^th^ and 5^th^ wk 65%, in the 6^th^ and 7^th^ wk 70%, in the 8^th^ wk 75%) |  |
| Ribas  (2017) | Experimental group | Virtual reality (Nintendo Wii) | - VR (30 min) : table tilt, tilt city, penguin slide, soccer heading, basic run, obstacle course, basic step | 30 minutes/day  2 days/week  12 weeks,  60-day follow-up |
|  | Control group | Conventional exercise | - PT (30 min) : warming, stretching and active exercises (10 min); resistance exercises for the limbs (10 min); and diagonal exercises for the trunk, neck and limbs (10 min). |  |
| Gandolfi  (2017) | Experimental group | Virtual reality  (Nintendo Tele Wii) | - VR (50 min) : table tilt, penguin slide, balance bubble, ski slalom, skateboarding, perfect 10, tilt city, snowball fight, rhythm parade, bird’s-eye bulls-eye | 50 minutes/day  3 days/week  7 weeks  1-month follow-up |
|  | Control group | Sensory integration balance training | - BT (50 min) : static weight bearing, trunk twist, postural transfers, dynamic weight bearing, external perturbed, unstable surfaces, Swiss ball, dual-task |  |
| Carpinella  (2017) | Experimental group | Gamepad-based training | - VR (45 min) : A set of balance and gait tailored exercises included within gamepad | 45 minutes/day  3 days/week  7 weeks,  1-month follow-up |
|  | Control group | Physiotherapy | - PT (45 min) : muscle stretching (hamstrings, quadriceps and calves), mobilization exercises (e.g. trunk rotation, hip abduction and flexion), balance and gait exercises |  |
| Yang  (2016) | Experimental group | VR balance training | - Warm-up stretching (10 min) - VR (30 min) : static posture maintaining (star excursion, home yoga), dynamic weight shifting (ball maze, table tilt, cooking, cloth washing, car racing, park walking, apple catching) - Break (10 min) | 50 minutes/day  2 days/week  6 weeks,  follow-up to 8 weeks |
|  | Control group | Conventional balance training | - Warm-up stretching (10 min) - BT (30 min) : static posture maintaining, dynamic weight shifting (choice stepping, rope crossing) - Break (10 min) |  |
| Shih  (2016) | Experimental group | Balance-based exergaming with Kinect | - Warm-up (10 min) - VR (30 min) : reaching task 1, reaching task 2, obstacle avoidance, marching - Cool-down (10 min) | 50 minutes/day  2 days/week  8 weeks |
|  | Control group | Conventional balance training | - Warm-up (10 min) - BT (30 min) : reaching activities, weight-shifting activities, marching activities - Cool-down (10 min) |  |
| Liao  (2015) | Experimental group | Virtual reality (Nintendo Wii) | - VR (45 min) : yoga exercises (10 min), strengthening exercises (15 min), balance games (20 min) | 45 minutes/day  2 days/week  6 weeks,  1-month follow-up |
|  | Control group | Traditional exercise | - PT (45 min) : stretching exercises (10 min), strengthening exercises (15 min), balance exercises (20 min) |  |
| van den Heuvel  (2014) | Experimental group | Visual feedback training | - VR (60 min) : leaning forward (downward movement and upward movement), functional tasks associated with standing balance | 60 minutes/day  2 days/week  5 weeks,  follow-up to 12 weeks |
|  | Control group | Conventional balance training | - BT (60 min) : training standing balance and included exercises while standing on one leg or with eyes closed, stepping exercises, dual-task exercises, sit to-stand exercises, and exercises on the balancing beam or other challenging support surfaces |  |
| Pedreira  (2013) | Experimental group | Virtual reality (Nintendo Wii) | - VR (40 min) : training on strength, balance, aerobics and boxing | 40 minutes/day  3 days/week  4 weeks |
|  | Control group | Physical therapy | - PT (40 min) : trunk and limb mobilization, balance, muscle strengthening, rhythmic movement, postural alignment, double-task execution, bimanual tasks, and cardiorespiratory and gait training |  |
| Pompeu  (2012) | Experimental group | Wii-based motor and cognitive training | - Global exercise (30 min) : stretching, strengthening and axial mobility exercises - VR (30 min) : static balance (single leg extension and torso twist), dynamic balance (table tilt, tilt city, soccer heading and penguin slide), stationary gait (rhythm parade, obstacle course, basic step and basic run). | 60 minutes/day  2 days/week  7 weeks  60-day follow-up |
|  | Control group | Global and balance exercise | - Global exercise (30 min) : stretching, strengthening and axial mobility exercises - Balance exercise (30 min) |  |

BE Bicycle exercise, BT balance training, MI Motor imagery, PT physical therapy, VR virtual reality
