# Supplementary material for: A systematic review and meta-analysis on the effect of virtual reality-based rehabilitation for people with Parkinson’s disease
Source: J Neuroeng Rehabil. 2023 Jul 20;20:94. doi: 10.1186/s12984-023-01219-3 (PMC10360300; doi:10.1186/s12984-023-01219-3)
Supplement: Supplementary file 4 — Additional file 4: Figure S1. Funnel plots for meta-analysis of balance function. Figure S2. Funnel plots for meta-analysis of gait ability. Figure S3. Funnel plots for meta-analysis of activities of daily living and motor function. Figure S4. Funnel plot for meta-analysis of quality of life. [file 12984_2023_1219_MOESM4_ESM.docx]

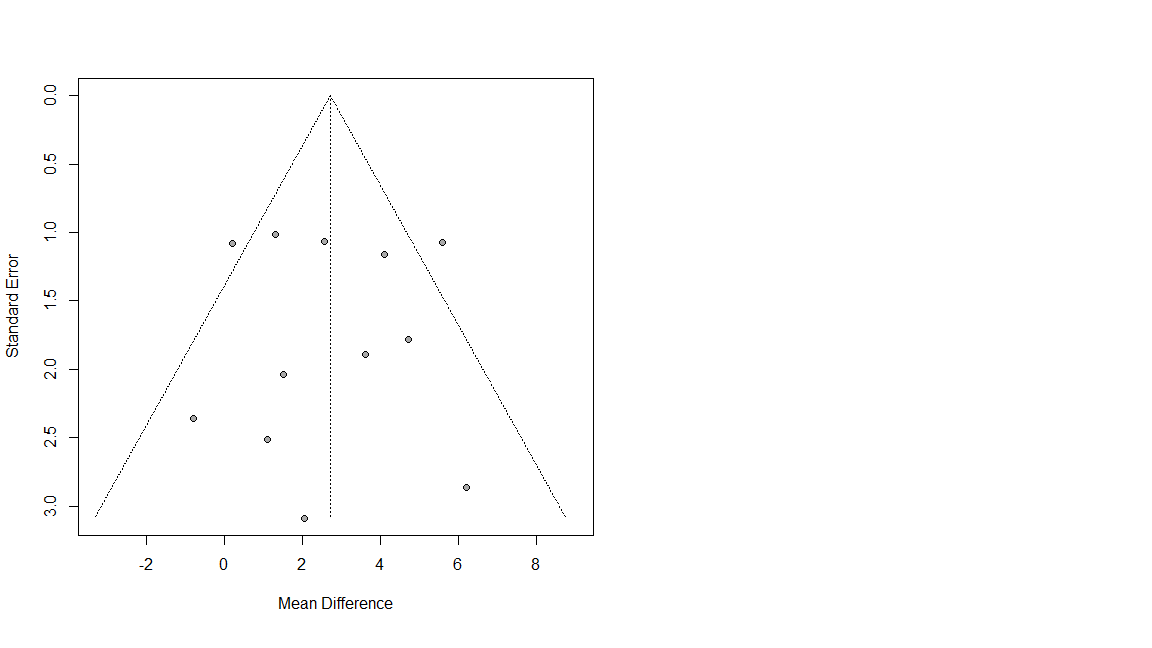


**b**

**a**


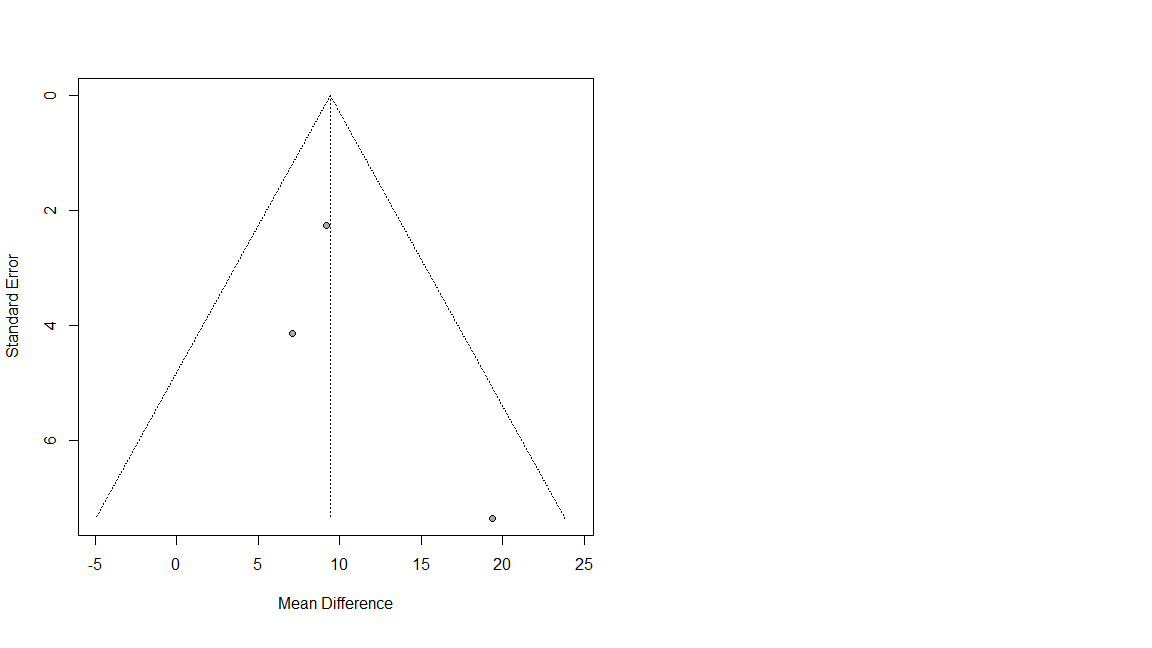


**c**


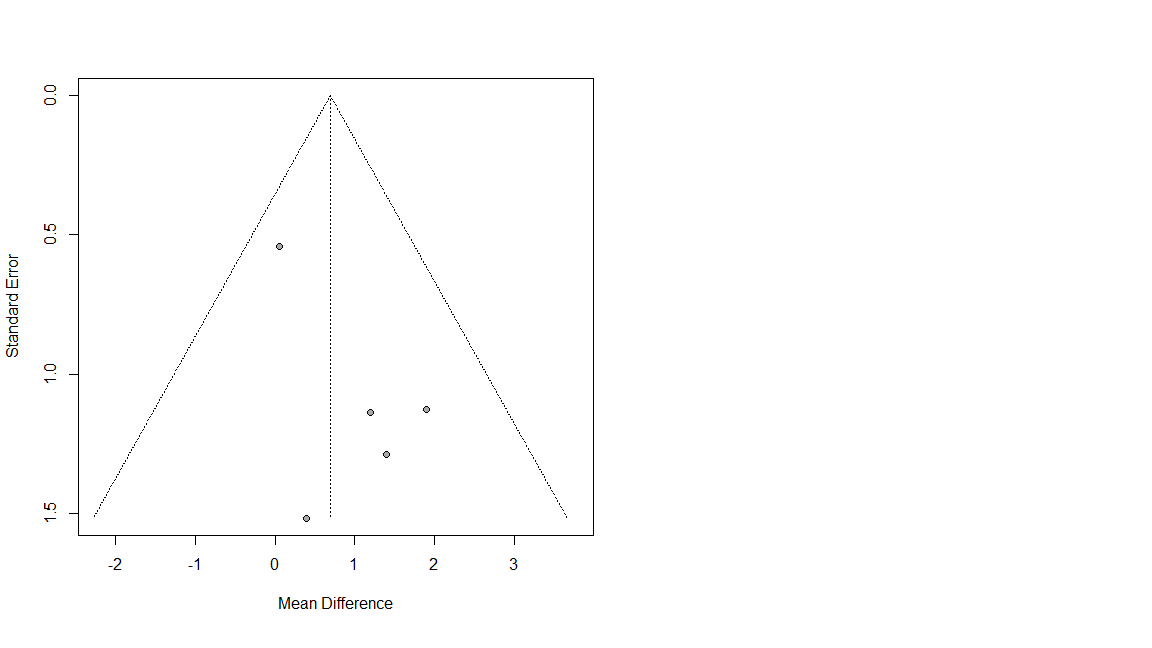


**Figure S1**. Funnel plots for the meta-analysis of balance function. (**a**) the Berg balance scale (BBS), (**b**) the activities-specific balance confidence (ABC), and (**c**) the dynamic gait index (DGI).


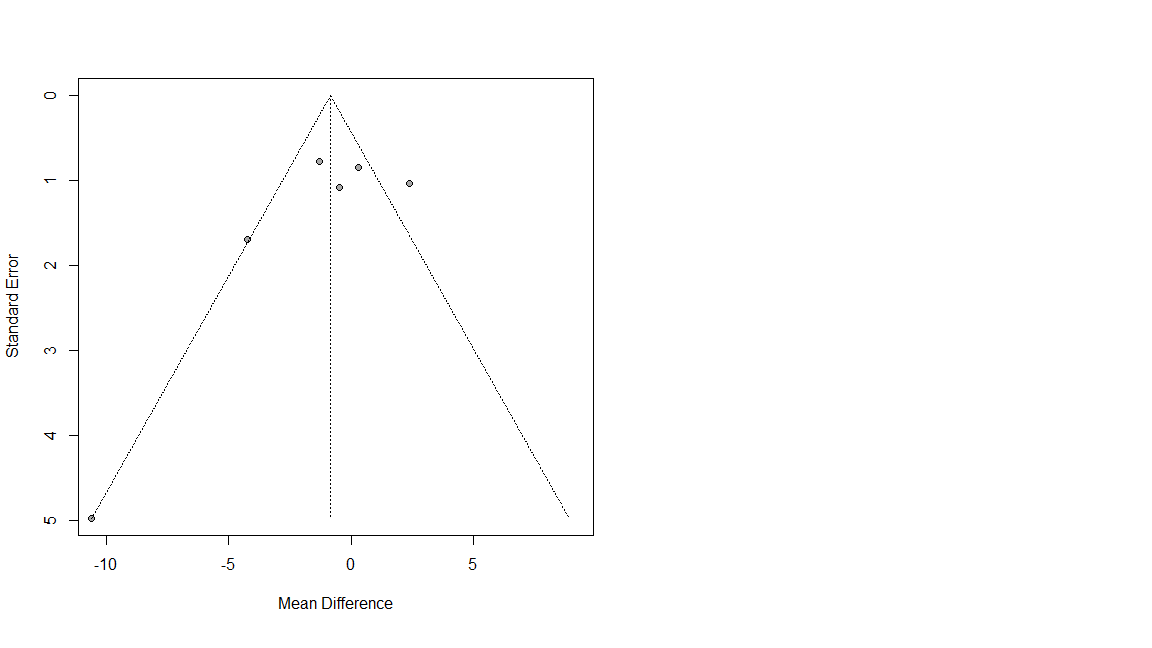


**a**


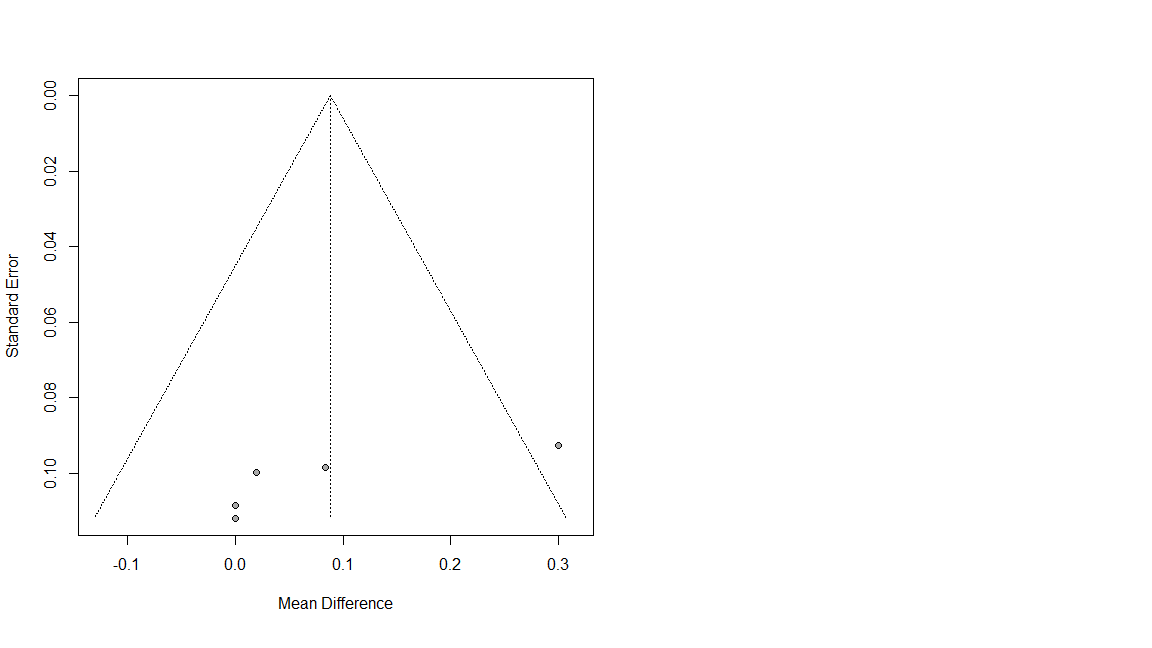


**b**


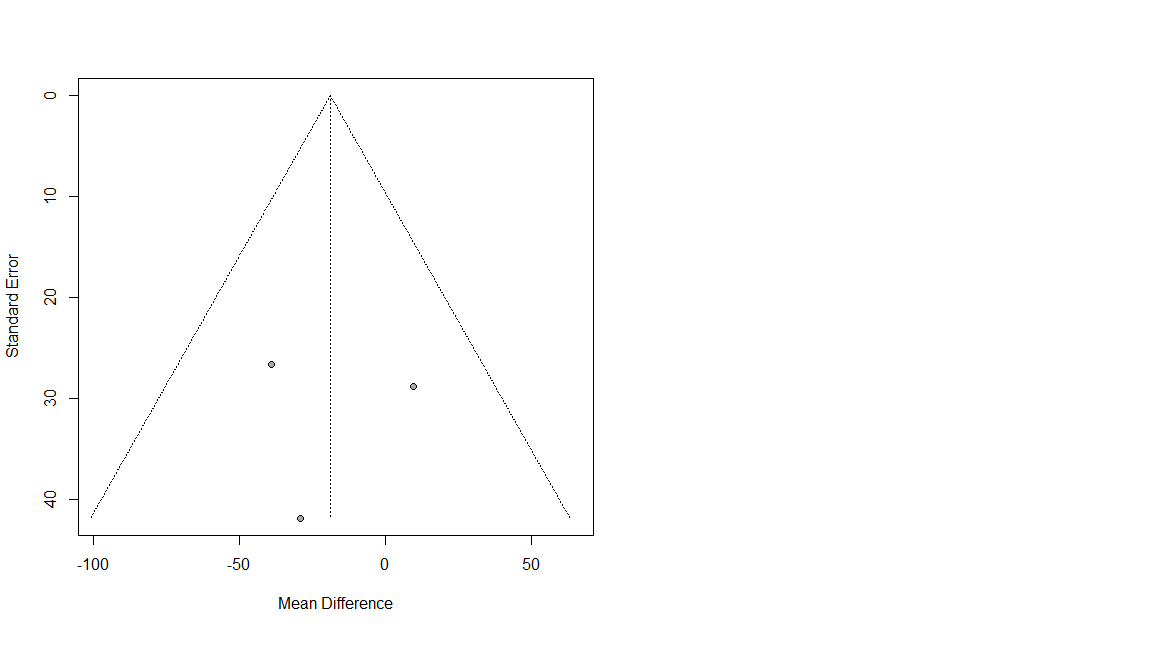


**c**

**Figure S2**. Funnel plots for the meta-analysis of gait ability. (**a**) the time up and go test (TUGT), (**b**) the 10-meter walk test (10MWT), and (**c**) the 6-minute walk test (6MWT).


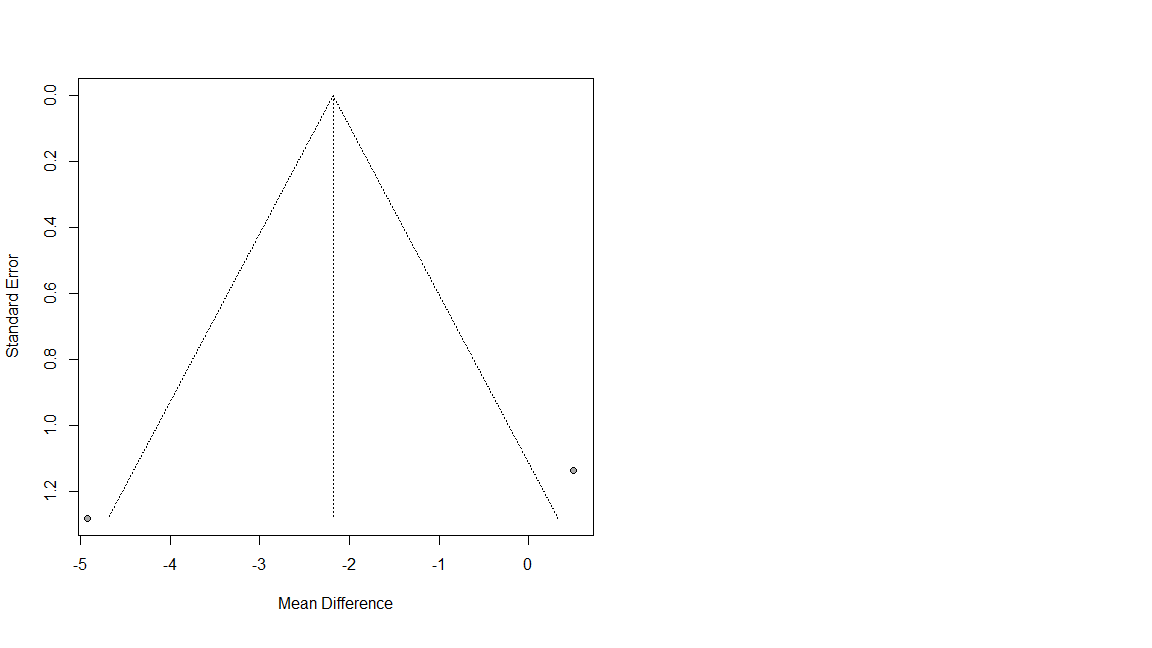


**a**


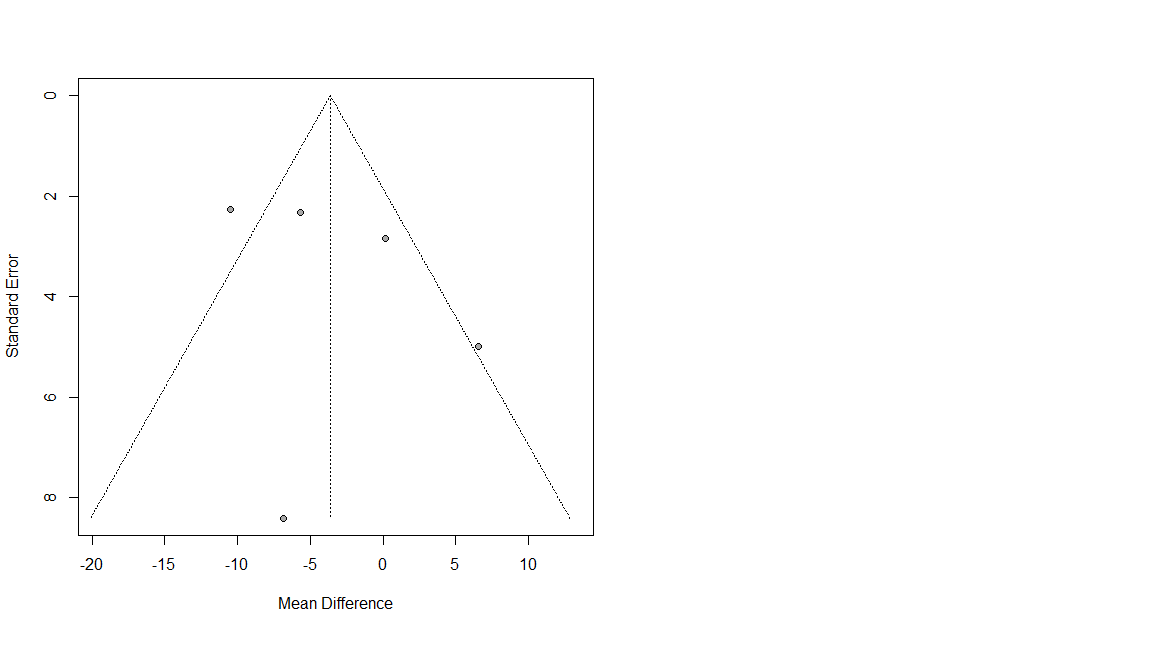


**b**

**Figure S3**. Funnel plots for the meta-analysis of activities of daily living and motor function. (**a**) the Unified Parkinson’s disease rating scale (UPDRS) II and (**b**) the UPDRS III.


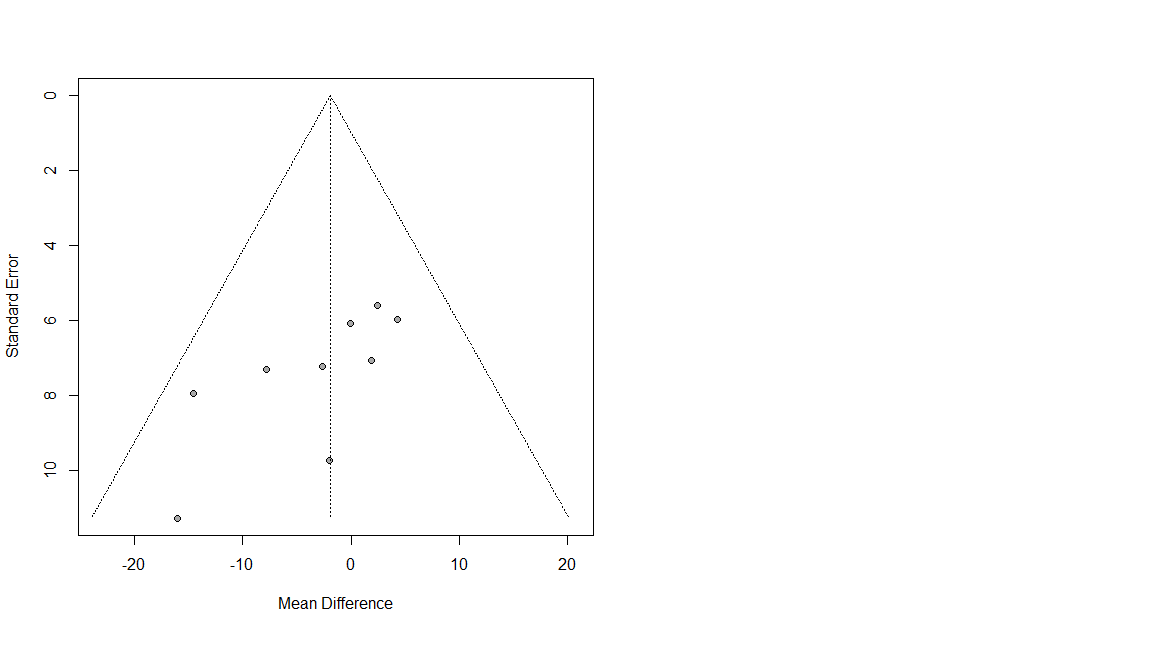


**Figure S4**. Funnel plot for the meta-analysis of quality of life measured by the Parkinson’s disease questionnaire-39 (PDQ-39).
